# New high intensity fibreoptic phototherapy devices in healthy newborns: a single pad wrapped around the neonate body in comparison with a double pad device

**DOI:** 10.1186/s13052-019-0663-5

**Published:** 2019-06-06

**Authors:** R. Luciano, G. Mancini, F. Cota, A. Romano, V. Purcaro, F. Lerro, M. Corsello, G. Vento

**Affiliations:** 1 Division of Neonatology, Department of Pediatrics, Fondazione Policlinico Universitario A. Gemelli IRCCS- Catholic University of Sacred Heart, Largo A. Gemelli, 8, 00168 Rome, Italy; 2 Catholic University of Sacred Heart, Rome, Italy

**Keywords:** Hyperbilirubinaemia, Jaundice, Newborn, Phototherapy

## Abstract

**Background:**

Fibreoptic Phototherapy (FPT) allows to lower total serum bilirubin (TSB) levels in healthy neonates maintained in rooming-in with their mothers. The 2004 Cochrane review showed that, differently from preterm neonates, FPT was not as effective as traditional phototherapy in term neonates (TN), unless the simultaneous utilization of two FPT devices. Aim of this study was to compare the efficacy of two FPT devices, both equipped with a single light-emitting diode (LED): the first one has a single large pad wrapped around the infant body (Bilisoft, GE Health Care) (device A), the second one is a double-pad phototherapy device (BiliCocoon, CremascolieIris) (device B).

**Methods:**

We studied 172 healthy neonates with non-hemolytic hyperbilirubinaemia: 57 TN and 57 late preterm neonates (LPN) treated with device A (Group 1); 47 TN and 11 LPN treated with device B (Group 2). We evaluated the differences between groups by the Student’s t-test for continuous variables and by chi square test for categorical data. A two tailed *p* < 0.05 was considered significant.

**Results:**

There were no differences in term of duration of FPT, TSB hourly reduction, percentage of TSB reduction after FPT, TSB maximum rebound, percentage of TSB increase after FPT discontinuation and number of after-discharge checks. Two neonates treated with device B showed no decrease in TSB values during FPT. Seven infants treated with device B experienced hyperpyrexia.

**Conclusions:**

The two LED FPT devices were both effective in lowering TSB either in TN or LPN. Device A was effective in all treated neonates without negative side effects during treatment; device B was effective in all but 2 infants and 12% of the neonates in the same group experienced hyperpyrexia. According to our results, the single big pad wrapped around the infant body has the same efficacy as a double FPT device in TN and LPN.

## Background

The 2004 AAP guidelines on management of neonatal jaundice focused on the opportunity “to reduce the incidence of severe hyperbilirubinaemia and bilirubin encephalopathy while minimizing the risks of unintended harm such as maternal anxiety, decreased breastfeeding and unnecessary costs or treatment” [1, 2]. Fibreoptic Phototherapy (FPT) devices, equipped with a pad placed directly in contact with the infant skin, allow to lower total serum bilirubin (TSB) levels in healthy neonates maintained in rooming-in with their mothers, avoiding interruption of exposure during feeding and separation of the mother-neonate dyad [3, 4]. The 2004 Cochrane review, however, showed that, differently from preterm neonates, FPT was not as effective as traditional phototherapy in term neonates (TN), unless two FPT devices were used simultaneously [5]. The new light emitting diode (LED) phototherapy device with its high luminous intensity, narrow wavelength band and higher irradiance could make phototherapy more efficacious than the previous phototherapy devices [6]. A previous study from our institution showed the efficacy of a new LED FPT device in lowering TSB levels in healthy term (TN) as well as in late preterm neonates (LPN) [7]. The new device consisted of a high intensity blue light lamp equipped with a large single pad wrapped around the infant body to increase the exposed skin surface. We hypothesized that this device was acting as a double phototherapy. To test this hypothesis, we compared, in the present study, the device equipped with the single pad wrapped around the infant’s body (Bilisoft, GE Health Care- Device A) with a device equipped with a double pad FPT device (BiliCocoon, CremascolieIris- Device B).

## Methods

We studied 172 healthy neonates submitted to FPT for non-hemolytic hyperbilirubinaemia during their hospital stay in rooming-in unit with their mothers.

Group 1 consisted of the hystorical group of 114 neonates (57 LPN and 57 TN) born between January 2013 and August 2016 treated with device A, a group already analyzed in our previous prospective study [7]. Group 2 was made up of 58 newborns (47 TN and 11 LPN), born between June 2017 and February 2018, who were submitted to FPT with device B. FPT was started according to the Italian Society of Neonatology guidelines, with different starting values for TN and LPN [8]. We checked TSB values after the first 6–8 h of treatment in order to establish the efficacy of FPT and every 8–12 h afterwards. Phototherapy was stopped in accordance with our NICU protocols including all the following: > 72 h of life, at least 24 h of FPT, constant decrease of TSB values (at least two consecutive values), TSB < 14 mg/dl in TN and < 12 mg/dl in LPN. We checked TSB rebound 12–24 h after stopping FPT, usually in post-discharge, and then every 24 h until a lower TSB value was obtained.

Group 1 and group 2 neonates were kept in 24 h Rooming-in with their mothers throughout their hospital stay, with a policy of breast-feeding on request. Infants’ body weight was controlled every day and the maximum weight loss was registered. Weight variation during phototherapy was calculated in the following way: (weight at start FPT – weight at stop FPT)/weight at start FPT *100. Daily urine output during FPT was evaluated through diaper weight and number of emissions.

Formula milk was offered to all preterm neonates submitted to FPT. A formula supply six times a day was also offered to term neonates if breastfeeding was not yet successfully established at the time when FPT was started. A percentage of 82% in Group 1 (47/57) and 80% (35/43) in Group 2 received formula during FPT. The daily amount of formula was not calculated, as it was left to the self-regulation of the newborn, depending on the amount of breast milk assumed before formula. The infant’s hydration status was assessed by daily weight and diuresis.

All babies included in the study passed meconium in the first 24 h of life.

Both FPT devices are equipped with high intensity blue LED light pads: device A consists of a single pad big enough to be wrapped around the infant body, device B is made up of a double pad more adherent to infant’s skin, acting as a double phototherapy. The pads were always settled on the naked neonates with the covers provided with both the devices. The two devices have the following technical features. Device A: light emission wavelength 430–490 nm; a single emitting surface size 25 × 30 cm; radiance: 50 microwatt/cm^2^/nm when directly applied on the naked neonate, 35 microwatt/cm^2^/nm, when the pad is correctly settled with the disposable cover provided with the device. Device B: light emission wavelength 430–490 nm, two light emitting surfaces size 20 × 30 cm, radiance 35 microwatt/cm^2^/nm ± 15% with the disposable cover provided with the device.

Axillary body temperature was strictly controlled during treatment. Hyperpyrexia was defined as body temperature ≥ 37.5 °C.

Criteria to switch to conventional phototherapy were the following: hyperpyrexia not resolved after placement of the arms outside the mat, infections or pathologies requiring closer monitoring of the vital parameters; hemolytic jaundice, failure of the FPT device in reducing bilirubin levels in two subsequent controls after phototherapy was started.

In order to demonstrate a statistically significant difference between the two FPT devices we decided to calculate the sample size on the hypothesis that Device B would be 50% more efficient than device A and we evaluated the treatment efficacy on the TSB hourly reduction. We chose to utilize the method used by Seidman et al., [9] who compared the efficacy of two different LED phototherapy devices (blue-green LEDS or blue LED) presuming that the former would be 50% more efficient than the latter on the basis of the hourly reduction of TSB. According with our previous study [7], where the mean rate of decrease in TSB in TN was 0.11 mg/dl/h with a Standard Deviation (SD) of 0.06, the expected mean rate of decrease in TSB with the use of device B was estimated as 0.16 mg/dl/h with a SD of 0.06. Setting a significance level of 0.05 and a power of 0.80, the calculated sample size required was determined to be 23 for each study group. Being the historic control group (Group A) represented by a sensible larger amount of neonates, we decided to organize the recruitment in order to reach a similar number in Group B.

TSB was measured using Ginevri One Beam Microbilimeter by determining absorbancy at 455 nm with correction for hemoglobin contamination (575 nm), coefficients of variation 1%.

FPT efficacy was evaluated as follows: TSB hourly variation during FPT, treatment duration, percentage of overall TSB reduction and percentage of TSB reduction after 24 h of FPT, TSB maximum rebound and number of after-discharge checks (Tables 1 and 2).

**Table 1:** Clinical characteristics of the study population and comparative effectiveness of FPT in TN

	TN group 1 (57)	TN group 2 (43)	*P*
Gestational age (wks)	38.6 ± 1.2	38.7 ± 1.2	0.68
Birthweight (g)	3288 ± 350	3277 ± 443	0.89
Gender (male)	31 (54.4)	25 (58,1)	0.71
AB0 mismatch	17 (29.8)	18 (41.9)	0.21
Rh mismatch	3 (5.3)	2 (4.6)	0.89
Cesarean section	7 (12.3)	4 (9.3)	0.64
Apgar score at 5’	9.7 ± 0.6	9.8 ± 0.4	0.51
SGA	0	0	
Age at FPT start (h)	63 ± 22	64 ± 21	0.89
TSB at FPT start (mg/dl)	15.4 ± 2.1	15.9 ± 1.8	0.18
TSB at FPT stop (mg/dl)	11.0 ± 1.1	11.1 ± 1.3	0.59
FPT duration (h)	47.6 ± 18.8	42.7 ± 16.8	0.18
TSB reduction at stop of FPT (%)	27.6 ± 10.4	29.0 ± 10.0	0.51
TSB hourly reduction in PT (mg/dl/h)	0.11 ± 0.06	0.14 ± 0.15	0.17
TSB maximum rebound (%)	11.1 ± 26.6	13.0 ± 19.0	0.69
Check numbers after discharge	1.9 ± 1.4	1.6 ± 1.2	0.24
Birthweight variation (%)	3.6 ± 4.2	3.8 ± 3.2	0.79
Weight variation during phototherapy (%)	−1.9 ± 5.2	−2.3 ± 2.2	0.60
TSB reduction after 24 h of FPT (%)	16.0 ± 9.0	18.0 ± 9.0	0.27

Data are expressed as mean ± SD or n. (%)

**Table 2:** Clinical characteristics of the study population and comparative effectiveness of FPT in LPN

	LPN group 1(57)	LPN group 2 (9)	*P*
Gestational age (wks)	35.1 ± 0.8	35.8 ± 0.4	0.02
Birthweight (g)	2657 ± 481	2603 ± 256	0.74
Gender (male)	26 (45.6)	5 (55.5)	0.58
AB0 mismatch	8 (14.0)	1 (11.1)	0.81
Rh mismatch	3 (5.3)	1 (11.1)	0.49
Cesarean section	15 (26.3)	0	0.09
Apgar score at 5’	9.2 ± 0.7	9.3 ± 0.5	0.59
SGA	2 (3.5)	1 (11.1)	0.31
Age at FPT start (h)	49 ± 17	55 ± 12	0.33
TSB at FPT start (mg/dl)	12.6 ± 2.0	13.6 ± 1.2	0.17
TSB at FPT stop (mg/dl)	8.9 ± 2.0	10.7 ± 1.5	0.01
FPT duration (h)	50.5 ± 18.4	50.9 ± 23.2	0.95
TSB reduction at stop of FPT (%)	28.9 ± 14.5	20.0 ± 16.0	0.10
TSB hourly reduction in PT (mg/dl/h)	0.08 ± 0.06	0.07 ± 0.07	0.65
TSB maximum rebound (%)	24.4 ± 20.2	28.0 ± 15.0	0.61
Check numbers after discharge	1.9 ± 1.2	2.2 ± 1.6	0.51
Birthweight variation (%)	3.7 ± 3.2	2.9 ± 1.9	0.47
Weight variation during phototherapy (%)	−0.2 ± 2.4	−0.8 ± 1.1	0.46
TSB reduction after 24 h of FPT (%)	15.0 ± 9.0	13.0 ± 11.0	0.55

Data are expressed as mean ± SD or n. (%)

Data are reported as mean ± standard deviation or count and percentage according to the nature of variables (continuous or count/categorical). The differences between groups were evaluated by the Student’s t-test for continuous variables; categorical data were analyzed by chi square test. A two tailed *p* < 0.05 was considered significant.

## Results

Tables 1 and 2 show the clinical characteristics and the FPT efficacy respectively in TN and LPN. No significant differences were found in the clinical characteristics between Group 1 and 2 either in TN or in LPN. Six neonates in Group 2 (4 TN and 2 LPN), among the 58 newborns initially enrolled, were excluded from the study. One neonate suffered from hemolytic hyperbilirubinaemia with positive Coombs test. Two neonates did not respond to the FPT with device B: Neonate 1: Gestational Age (GA) 37 weeks, phototherapy was started at 28 h of life with a TSB value of 11 mg/dl. After 37 h of phototherapy, TSB value was 14.8 mg/dl with two subsequent increases of TSB. Neonate 2: GA 37 weeks, phototherapy was started at 111 h of life with a TSB value of 18 mg/dl. After 43 h of phototherapy, TSB value was 16.4 mg/dl with two subsequent increases of TSB. The neonates were transferred to the department of Neonatal Pathology, in order to start conventional phototherapy in combination with FPT. Both neonates had no underlying clinical problems: urinary tract infections, G6PD deficiency, spherocitosis and hypothyrodism were excluded. Patient 1, GA 37 weeks, had an early TSB rise (FPT was started early on the second day of life: 28 h), differently from the other TN (Group 1: 63 ± 22 h; Group 2: 64 ± 21 h). Patient 2 was exclusively breastfed and had a pronounced weight loss concomitantly with a secondary TSB rise. The treatment with the combined phototherapy was successful in both neonates.

Two neonates were transferred in the department of Neonatal Pathology for hyperpyrexia (1 of them had urine culture positive for *Enterococcus faecium*), and one neonate for electrocardiographic signs of right heart hypertrophy.

FPT was started at 63 ± 22 h of life in TN of group 1 and 63 ± 21 h in TN of group 2 (p 0.89); it was started earlier in LPN, at 49 ± 17 h in group 1 and 55 ± 12 h in group 2 (p 0.33). Duration of FPT was 47.58 ± 18.82 h in TN and 50.49 ± 18.37 in LPN in Group 1, in comparison with 42.67 ± 16.77 (*p* = 0.18) and 50.89 ± 23.22 (*p* = 0.95) respectively in Group 2. TSB hourly reduction was 0.11 ± 0.06 in TN and 0.08 ± 0.06 in LPN of Group 1 vs 0.14 ± 0.15 (*p* = 0.17) and 0.07 ± 0.07 (*p* = 0.65) respectively in TN and LPN of Group 2. The percentage of TSB reduction after treatment with device A was 27.64 ± 10.39 in TN and 28.86 ± 14.51 in LPN, while it was 29 ± 10 in TN (*p* = 0.51) and 20 ± 16 in LPN (*p* = 0.10) with device B.

The percentage of TSB reduction at 24 h of treatment was: 16 ± 9% in TN of Group 1 and 18 ± 9% in TN of Group 2 (p 0.27); it was 15 ± 9% in LPN of Group 1 and 13 ± 11% in LPN of Group 2 (p 0.55).

There is a significant difference between group 1 and 2 regarding TSB values at FPT discontinuation among LPN. This data could be the consequence of the low number of LPN we enrolled in group 2, due to the usual lower percentage of LPN birth in respect of TN births .

After stopping FPT, TN treated with device A showed 11.09 ± 26.65% rebound vs 13 ± 19% (*p* = 0.69) with device B. This percentage was 24.4 ± 20.2% vs 28 ± 15% (*p* = 0.61) in LPN. The maximum rebound occurred 24–72 h after FPT discontinuation in both groups. Both TN and LPN needed a similar number of TSB controls after discharge not different in the 2 groups: 1.88 ± 1.43 (TN) and 1.91 ± 1.24 (LPN) in Group 1, 1.56 ± 1.22 (TN, *p* = 0.24) and 2.22 ± 1.64 (LPN, *p* = 0.51) in Group 2.

There were no differences in maximum weight loss and weight variation during FPT in the two groups.

FPT was easily managed in the rooming-in unit without interference with breastfeeding and with good mothers’ compliance.

Seven babies in Group 2 experienced hyperpyrexia, which recovered displacing arms outside the pad.

## Discussion

FPT makes it possible to treat hyperbilirubinaemia in neonates maintained in rooming-in with their mothers, avoiding interference with breastfeeding and separation of the mother-neonate dyad [3, 4]. The efficacy of phototherapy relays on multiple factors: wavelength, irradiance, exposed body surface area, distance of the phototherapy from the neonatal body, and duration of exposure [1].

Device A FPT has a radiance of 50 microwatt/cm^2^/nm when directly applied on the naked neonate, but the actual radiance stated by the technical specifications is 35 microwatt/cm^2^/nm, when the pad is correctly settled with the disposable cover provided with the device. Device B radiance is calculated by the factory to be 35+ 5 microwatt/cm^2^/nm, with the pad folded in the disposable cover provided with the device. Both devices provide a radiance that is in accordance with the AAP recommendations for intensive phototherapy, that imply a radiance of 30 μW/cm2/nm or higher in the 430 to 490 nm band, delivered to the greatest body surface area. Radiance higher than 30–35 microwatt/cm^2^/nm produces no added efficacy, probably for the existence of a saturation point. [1]

A light-emitting diode (LED) is a new type of light source that emits more intense narrow bandwidth blue light when compared to a fibreoptic device with a halogen light source. There are evidences that LED phototherapy is efficacious in bringing down levels of serum total bilirubin at rates similar to phototherapy with conventional (CFL or halogen) light sources [6, 10, 11]. There are no studies comparing the efficacy of LED fibreoptic phototherapy with conventional or fibreoptic non-LED phototherapy. Phototherapy efficacy has been evaluated with different methods in different studies mostly including hourly TSB reduction during treatment [3, 4, 9, 10, 12, 13] duration of treatment [14, 15] and the absolute and relative change in TSB levels during phototherapy [16–18] . We evaluated the treatment efficacy considering TSB hourly reduction during FPT, treatment duration, percentage of overall TSB reduction after FPT, TSB maximum rebound and number of after-discharge checks. We also evaluated the percentage of TSB reduction after 24 h of treatment and no statistically significant difference between the two devices was observed.

The efficacy of device A was not different from device B in phototherapy duration, TSB hourly reduction and TSB percentage reduction after treatment. However, two neonates did not respond to the FPT with device B and continued their treatment with conventional phototherapy. On the contrary, device A FPT was efficient in lowering TSB values in all treated neonates.

Moreover, hyperpyrexia was found in 12% of Group 2 neonates, in contrast with no hyperthermia episode in group 1 neonates. The hyperthermia observed in group 2 neonates might be related to the tight adherence of the double pad allowing a lower air passage through the mat. A study conducted by Ng and colleagues [19] showed a statistically detectable increase in axillary temperature occurring after 90 min of phototherapy with BiliSoft LED Phototherapy System (GE Health Care) in infants at post-conceptional age > 34 + 0 weeks: axillary temperature was greater than 37.5 °C in 4 babies among 29 (13.8%).

The 2004 Cochrane review, showed that, differently from preterm neonates, FPT was not as effective as traditional phototherapy in term neonates (TN), unless two FPT devices were used simultaneously [5].

Our previous study showed that high intensity LED fibreoptic device equipped with a large pad wrapped around the infant body, resulted strongly effective in lowering TSB levels in healthy TN as well as in LPN neonates [7].

The present study shows that a LED fibreoptic device equipped with a pad wrapped around the neonate body to increase the exposed body surface shows similar TSB hourly reduction, similar FPT duration and overall TSB percentage reduction than a two pad (double phototherapy) LED fibreoptic device. These results were obtained in all treated Group 1 neonates, without the side effects (hyperthermia) observed in Group 2 neonates, where 2 neonates did not respond to treatment.

The major limitation of our study is having compared a series of patients treated with device B with an historical group of patients treated with the device A, a group already analyzed in our previous prospective study [7]. It is noteworthy, however, that the clinical setting and the clinical procedures underwent no changes between the two periods. The small number of LPN included in Group 2 is also to be taken into account when looking at the results in this gestational age group.

## Conclusions

The single big pad wrapped around the infant body acts as a double FPT device, being effective in all cases without side effects. The device B was effective in all but two infants and hyperpyrexia was an important side effect.

## Data Availability

The datasets used and/or analysed during the current study are available from the corresponding author on reasonable request.

## Abbreviations

FPT: Fibreoptic Phototherapy
LED: Light-emitting diode
LPN: Late preterm neonates
TN: Term neonates
TSB: Total serum bilirubin
